# *Chlamydia trachomatis*-specific T Cell Immunity Reflects Widespread Exposure in South African Adolescents and Young Women

**DOI:** 10.1093/infdis/jiaf595

**Published:** 2025-12-03

**Authors:** Rubina Bunjun, Micaela Lurie, Smritee Dabee, Shaun Barnabas, Venessa Maseko, Shameem Z Jaumdally, Hoyam Gamieldien, David A Lewis, Heather B Jaspan, Katherine Gill, Linda-Gail Bekker, Jo-Ann S Passmore

**Affiliations:** Institute of Infectious Disease and Molecular Medicine, University of Cape Town, Cape Town, South Africa; DSI-NRF CAPRISA Centres of Excellence in HIV Prevention, Durban, South Africa; Division of Medical Virology, Department of Pathology, University of Cape Town, Cape Town, South Africa; Institute of Infectious Disease and Molecular Medicine, University of Cape Town, Cape Town, South Africa; DSI-NRF CAPRISA Centres of Excellence in HIV Prevention, Durban, South Africa; Division of Medical Virology, Department of Pathology, University of Cape Town, Cape Town, South Africa; Institute of Infectious Disease and Molecular Medicine, University of Cape Town, Cape Town, South Africa; DSI-NRF CAPRISA Centres of Excellence in HIV Prevention, Durban, South Africa; Division of Medical Virology, Department of Pathology, University of Cape Town, Cape Town, South Africa; Institute of Infectious Disease and Molecular Medicine, University of Cape Town, Cape Town, South Africa; Desmond Tutu HIV Centre, University of Cape Town, Cape Town, South Africa; Department of Paediatrics and Child Health, Family Centre for Research with Ubuntu, Stellenbosch University, Stellenbosch, South Africa; National Health Laboratory Service, Centre for HIV and STIs, National Institute for Communicable Diseases, Johannesburg, South Africa; Institute of Infectious Disease and Molecular Medicine, University of Cape Town, Cape Town, South Africa; Division of Medical Virology, Department of Pathology, University of Cape Town, Cape Town, South Africa; Institute of Infectious Disease and Molecular Medicine, University of Cape Town, Cape Town, South Africa; Division of Medical Virology, Department of Pathology, University of Cape Town, Cape Town, South Africa; Division of Medical Virology, Department of Pathology, University of Cape Town, Cape Town, South Africa; Western Sydney Sexual Health Centre, Western Sydney Local Health District, Parramatta, New South Wales, Australia; Sydney Institute for Infectious Diseases and Westmead Clinical School, University of Sydney, Sydney, New South Wales, Australia; Institute of Infectious Disease and Molecular Medicine, University of Cape Town, Cape Town, South Africa; Seattle Children's Research Institute and University of Washington, Seattle, USA; Desmond Tutu HIV Centre, University of Cape Town, Cape Town, South Africa; Institute of Infectious Disease and Molecular Medicine, University of Cape Town, Cape Town, South Africa; Desmond Tutu HIV Centre, University of Cape Town, Cape Town, South Africa; Institute of Infectious Disease and Molecular Medicine, University of Cape Town, Cape Town, South Africa; DSI-NRF CAPRISA Centres of Excellence in HIV Prevention, Durban, South Africa; Division of Medical Virology, Department of Pathology, University of Cape Town, Cape Town, South Africa; National Health Laboratory Services, Cape Town, South Africa

**Keywords:** Chlamydia trachomatis, T cells, cytokines, genital tract, serology

## Abstract

**Background:**

*Chlamydia trachomatis* remains the most prevalent bacterial sexually transmitted infection worldwide, disproportionately affecting adolescent girls and young women (AGYW). Vaccine development is hindered by a limited understanding of protective immunity, particularly in the context of multiple exposures and immunopathology.

**Methods:**

We characterized mucosal inflammation and systemic immune responses to *C. trachomatis* in South African AGYW (n = 145), stratified by exposure history based on nucleic acid amplification testing (NAAT) and serology (Ab). Specifically, cervicovaginal cytokines, cervical T cell activation, and C. trachomatis-specific CD4+ T cell responses were assessed.

**Results:**

A NAAT+/Ab + status, signifying untreated/recurrent infection, was associated with increased cervical T cell activation. These women all had detectable *C. trachomatis*-specific CD4+ T cells in blood; however, the magnitude of the response was 2.4-fold lower than in NAAT−/Ab + (cleared infection) or NAAT+/Ab− (primary infection) groups. *C. trachomatis*-specific multifunctional CD4+ T cells were highest in NAAT−/Ab + women, and nearly absent in those who were NAAT+/Ab +. Notably, systemic *C. trachomatis*-specific Th1 responses were overall inversely correlated with genital tract concentrations of inflammatory cytokines, including IL-1β, TNF, and IL-17A.

**Conclusions:**

Both the magnitude and quality of the systemic CD4+ T cell responses are critical components of protective immunity to *C. trachomatis* and may limit mucosal immunopathology, informing vaccine strategies in high-risk populations.

## Teaser

*C. trachomatis*-specific Th1 responses were associated with lower mucosal inflammation, while exposure was associated with fewer polyfunctional Th1 cells. Findings suggest that both the magnitude and quality of T cell responses are key requirements for protective immunity and limiting immunopathology.

*Chlamydia trachomatis* is the most prevalent bacterial sexually transmitted infection (STI) globally. In 2020, the World Health Organization (WHO) estimated that there were more than 120 million new infections worldwide [1]. Adolescent girls and young women (AGYW), in particular, experience a high chlamydia burden [2]. Although the interruption in sexual healthcare delivery caused by the COVID-19 pandemic contributed to an increase in infections [3], chlamydia prevalence was already increasing in 2019 [4, 5]. Despite the availability of effective antibiotic treatment, many infections remain unnoticed and untreated [6], particularly in low-and-middle income countries (LMICs), which rely on the syndromic management for STIs [7, 8]. The WHO has set a target of a 50% reduction in new cases of chlamydia by 2030 [1]. Achieving this objective requires a prevention-oriented strategy, with an emphasis on vaccines [9].

Chlamydia vaccine development has been hindered by several immune-related obstacles [10, 11]. Live-attenuated vaccines carry the risk of inducing genital tract pathology [12], which is associated with pelvic inflammatory disease and subsequent infertility [13, 14] and may increase the risk of HIV-1 acquisition [15]. Therefore, an effective vaccine needs to induce a protective immune response without the accompanying mucosal immunopathology. Furthermore, determining correlates of protection is complicated by the natural history of *C. trachomatis* infection. Untreated or recurrent infections are common in young women but decrease with age, suggesting some immune protection after exposure, although incomplete [16–19].

CD4+ T cells play a key role in immunity against chlamydia, primarily due to their production of Th1 cytokines [20–23]. Clinical studies have focused on antigen mapping, with limited data characterizing the T cell response to *C. trachomatis*, especially in high-risk young women or in relation to exposure history [24–26]. Consequently, there is an urgent need to better understand the development of immunity while also considering the development of immune-mediated pathology due to multiple exposures. We characterized genital tract inflammation and systemic CD4+ T cell responses to *C. trachomatis* in South African AGYW, stratified by their infection history and relative exposure levels, to better understand immune correlates of prior cleared infections, and the effect of continuous pathogen exposure.

## METHODS

### Study Participants

AGYW (16–22 years old) from South Africa were enrolled in the Women's Initiative in Sexual Health (WISH) study and provided samples every 2 months for 6 months [27, 28]. A total of n = 149 AGYW completed the baseline study visit, 127/149 completed 2 visits, and 88/149 completed 3. Participants were excluded if they were living with HIV, pregnant, menstruating at the time of sampling, or if they were sexually active, douched, or used spermicides in the last 2 days or took antibiotics in the last 2 weeks. The Human Research Ethics Committee of the University of Cape Town (267/2013) approved the study.

### Study Procedures

Samples were collected at each study visit [28, 29]. A vulvovaginal swab was collected for STI testing, and cervicovaginal secretions (CVSs) were collected through insertion of a menstrual cup (Softcup; The Flex Company, USA) into the vagina for 30 minutes. Participants were tested for STIs at every visit. Specimens positive for *C. trachomatis* were also tested for lymphogranuloma venereum (LGV)-associated L1-L3 genovars. All participants were negative for LGV. Participants who had signs/symptoms of STIs or who tested positive were offered treatment and a partner referral letter.

### Sample Processing

Peripheral blood mononuclear cells (PBMC) and plasma were isolated by density gradient centrifugation with Histopaque (Sigma Aldrich, USA) within 4 hours of collection. Cells were cryopreserved in fetal calf serum (HyClone; Cytiva, USA) with 10% DMSO (Sigma-Adrich, USA) and stored in liquid nitrogen until use. Plasma was aliquoted and stored at −80°C. CVSs were removed from the menstrual cup as previously described [28, 30], diluted 1:5 with phosphate-buffered saline (PBS; Sigma Aldrich, USA), aliquoted, and stored at −80°C.

### Stimulation of T Cells

We selected participants with a positive *C. trachomatis* nucleic acid amplification test (NAAT) or who were seropositive for *C. trachomatis* at any visit during the 6-month study period, where samples were available (n = 46). Cryopreserved PBMC from the baseline visit were thawed and rested in R10 medium (Gibco RPMI 1640; Thermo Fisher Scientific, USA), with the addition of 10% heat-inactivated FCS (HyClone; Cytiva, USA) and 50 U/mL of penicillin-streptomycin (Sigma-Aldrich, USA) for 4 hours prior to stimulation. Cells were incubated in R10 with rMOMP protein (10μg/mL; Uniprot Accession #P17451.1, ProMab, USA) for 18 hours at 37°C in the presence of the costimulatory antibodies anti-CD28 and anti-CD49d (1μg/mL each; BD Biosciences, USA) and Brefeldin A (BFA; 5μg/mL, Sigma-Aldrich).

### Multiparameter Flow Cytometry

Stimulated PBMC were stained with a violet viability dye (ViViD; Molecular Probes, USA), followed by staining with CD14-Pacific blue, CD19-Pacific blue, CD4-PE-Cy5.5 (all Invitrogen, USA), CD8-BV711 (Biolegend, USA), CD27-PE-Cy-5 (Beckman Coulter, USA), and CD45RO-BV785 (Biolegend, USA). Cells were permeabilized with Cytofix-Cytoperm (BD Biosciences, USA), stained intracellularly with CD3-APC-H7, IFN-γ-Alexa700, IL-2-APC (all from BD Biosciences, USA), and TNF-α-PE-Cy7 (Biolegend, USA), and fixed with CellFix (BD Biosciences, USA). Cells were acquired on a BD LSRII (BD Biosciences, USA), using FACSDiva software. Data were analyzed using FlowJo v10 (TreeStar, BD Biosciences, USA) and Pestle and Spice [31]. The gating strategy is shown in Supplementary Figure 1. A positive cytokine response was defined as at least double the background frequency of the unstimulated sample and a net response >0.005%. All data are reported after background subtraction.

### Measurement of *C. trachomatis*-specific IgG by ELISA

Baseline visit plasma from 145 women was used to measure IgG using a semiquantitative ELISA kit licensed for diagnostic use with 100% sensitivity and 97% specificity (Cat#EI2191-9601G; EuroImmun; Germany). Plasma was diluted and incubated on the supplied precoated plate. The plate was washed and incubated with peroxidase-labelled anti-IgG, followed by a final incubation with tetramethylbenzidine (TMB) substrate. Stop solution was added, and color intensity was measured at 450 and 650 nm (reference) within 15 minutes. A standard curve was used to calculate the relative units (RU) of IgG. Results of >22 RU/mL were positive, 16–22 RU/mL were borderline, and <16 RU/mL were considered negative. Trachoma (serovars A-C) is not endemic to South Africa, and all participants were negative for LGV, suggesting the IgG detected arose from *C. trachomatis* serovar D-K infections. Similarly, cross-reactive antibodies generated by *C. pneumoniae* are unlikely due to their rarity in South Africa [32, 33]. While the derivative serovar(s) of the rMOMP used in the kit are not disclosed, the manufacturer states that the ELISA detects all serovars of human *C. trachomatis* and is not cross-reactive for human infection with *C. pneumoniae* or *C. psittaci*.

### Cervicovaginal Cytokine Assays

Diluted CVSs were thawed and filtered through a 0.2μm cellulose acetate filter. The Human Cytokine Group I 27-plex and Human Cytokine 21-plex kits (Bio-Rad, USA) were used to quantify a range of 44 cytokines, chemokines, and growth factors. Values below the detection limit were recorded as half of the lowest measured concentration for each cytokine. IL-2, IL-5, IL-15, and RANTES were excluded for all analyses as the values did not pass QC or >50% of values were below the detection limit.

### Statistical Analyses

Statistical analyses were performed using Prism 9 (GraphPad, USA). Nonparametric tests (Mann-Whitney U, Wilcoxon matched pairs, and Spearman Rank) were used for comparisons. Fischer's exact test was used to compare proportions of responders. A *P* value of <.05 was considered statistically significant.

## RESULTS

### Cohort Description

We used baseline samples from a well-characterized cohort of HIV-uninfected AGYW (median age of 18 years old) from South Africa [27, 28]. Participants were recruited within 3 years of sexual debut and reported a median of 2 sexual partners (Table 1). Hormonal contraceptive use was high (n = 143/145), but only 34% (42/122) of participants reported regular condom use within the past year. Importantly, a high prevalence of chlamydia (43%) was found, with low rates of previous STI diagnosis or treatment (14%), highlighting the need for better sexual health interventions.

**Table 1.** Participant characteristics

Characteristic	N = 145
Age in y [median (IQR)]	18 (17–20)
Age of sexual debut (y) [median (IQR)]	16 (15–17)
Lifetime sexual partners [median (range)]	2 (1–13)
Multiple sexual partners in the last 12 m [n/N(%)]	33/111 (30%)
Contraceptive use [n/N (%)]	
Ever pregnant	30/122 (25%)
Previous contraceptive use	96/122 (79%)
Condom use during the last sex act	87/121 (72%)
Regular condom use in the last 12 m	42/122 (34%)
Hormonal contraceptives	
DMPA-IM	27/145 (19%)
Net-En	99/145 (68%)
Combined oral contraceptive	7/145 (5%)
LNG implant	9/145 (6%)
Nuvaring	1/145 (0.7%)
Sexually transmitted infections (STIs) [n/N (%)]	
*Chlamydia trachomatis*	62/145 (43%)
*Neisseria gonorrhoeae*	17/145 (12%)
*Mycoplasma genitalium*	6/145 (4%)
*Trichomonas vaginalis*	11/145 (8%)
Bacterial vaginosis (Nugent score 7–10)	70/145 (48%)
Previous STI (diagnosed or treated)	17/122 (14%)

### Classification of *C. trachomatis* Infection History and Exposure Levels Using Nucleic Acid Amplification Testing and Serology

**Figure 1.**
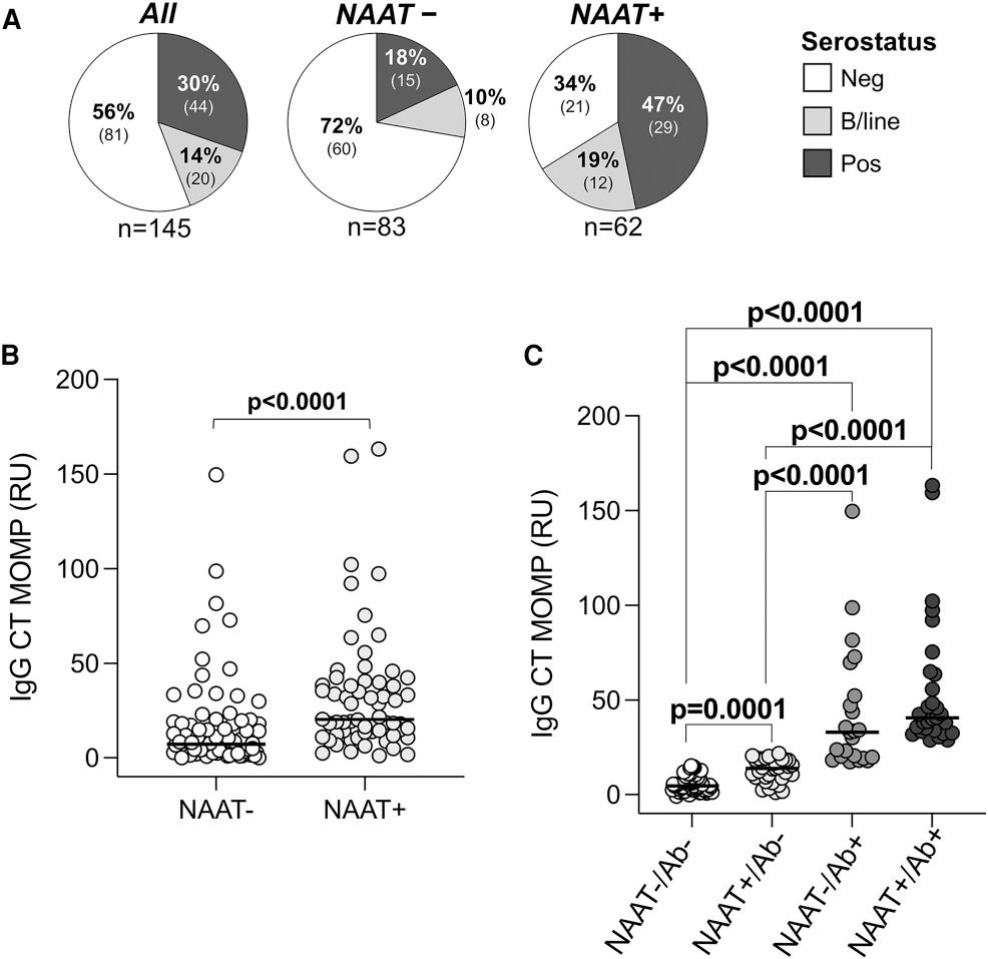
Repeated or persistent *C. trachomatis* infections were common in adolescent girls and young women (n = 145). (*A*) Plasma serology results from the cohort were measured using semi-quantitative ELISA (Euroimmun; Germany). Relative units (RU) were used to classify participants into negative (<16 RU; neg; white), borderline positive (16–21 RU; b/line; light grey), and positive (≥22 RU; pos; dark grey). (*B*) Distribution of IgG levels measured by ELISA. (*C*) IgG levels stratified by exposure. Exposure groups were assigned based on NAAT results and serostatus. Each symbol represents an individual. Data were analyzed using a nonparametric Mann-Whitney *U* test. *P* < .05 was considered statistically significant. Ab = serostatus; CT = *C. trachomatis*; MOMP = major outer membrane protein; NAAT = nucleic acid amplification test.

We used anti-*C. trachomatis* IgG serology to infer infection history. Thirty percent (30%; 44/145) of participants were seropositive, 14% (20/145) were borderline positive, and 56% (81/145) were seronegative (Supplementary Table 1). Of those with a negative nucleic acid amplification test (NAAT), 72% (60/83) were also seronegative (Figure 1*A*). Similarly, 66% (41/62) of NAAT + AGYW were also seropositive. Notably, 34% (21/62) NAAT + AGYW were seronegative, likely signifying a primary infection. Women who were NAAT + also had nearly 3-fold higher antibody levels than women who were NAAT− (Figure 1*B*; *P* < .0001; medians: 20.35RU and 7.14RU, respectively), suggesting higher antigen load due to an active infection [34]. Given the low prevalence of previous STIs, and the asymptomatic nature of infections in this cohort, it is probable that NAAT + AGYW were undiagnosed and untreated, rather than having treatment failure (persistence) or repeated infections (recurring).

We next estimated the level of *C. trachomatis* exposure in this cohort, based on NAAT and serology (Supplementary Table 1). NAAT−/Ab− participants were uninfected, with no evidence of prior or current infection. AGYW with the lowest relative exposure were NAAT+/Ab− and likely had an early, primary infection prior to the development of antibodies [35]. Since antichlamydia antibodies can persist for at least 10 years [34, 36], NAAT−/Ab + participants had previously cleared or treated infections. The group with the highest exposure, NAAT+/Ab + participants, was considered to have an untreated infection of unknown duration or less likely, a recurrent (reinfection) or persistent (treatment failure) infection [36, 37]. Antibody levels increased progressively across exposure groups, with median IgG values of 4.61RU, 13.85RU, 32.98RU, and 40.69RU, respectively (Figure 1*C*). This grouping framework enabled a nuanced assessment of exposure history in a highly affected cohort and provided a unique window into the development of immune responses during early *C. trachomatis* infection.

### *C. trachomatis* Infection History and Genital Tract T Cell Activation

**Figure 2.**
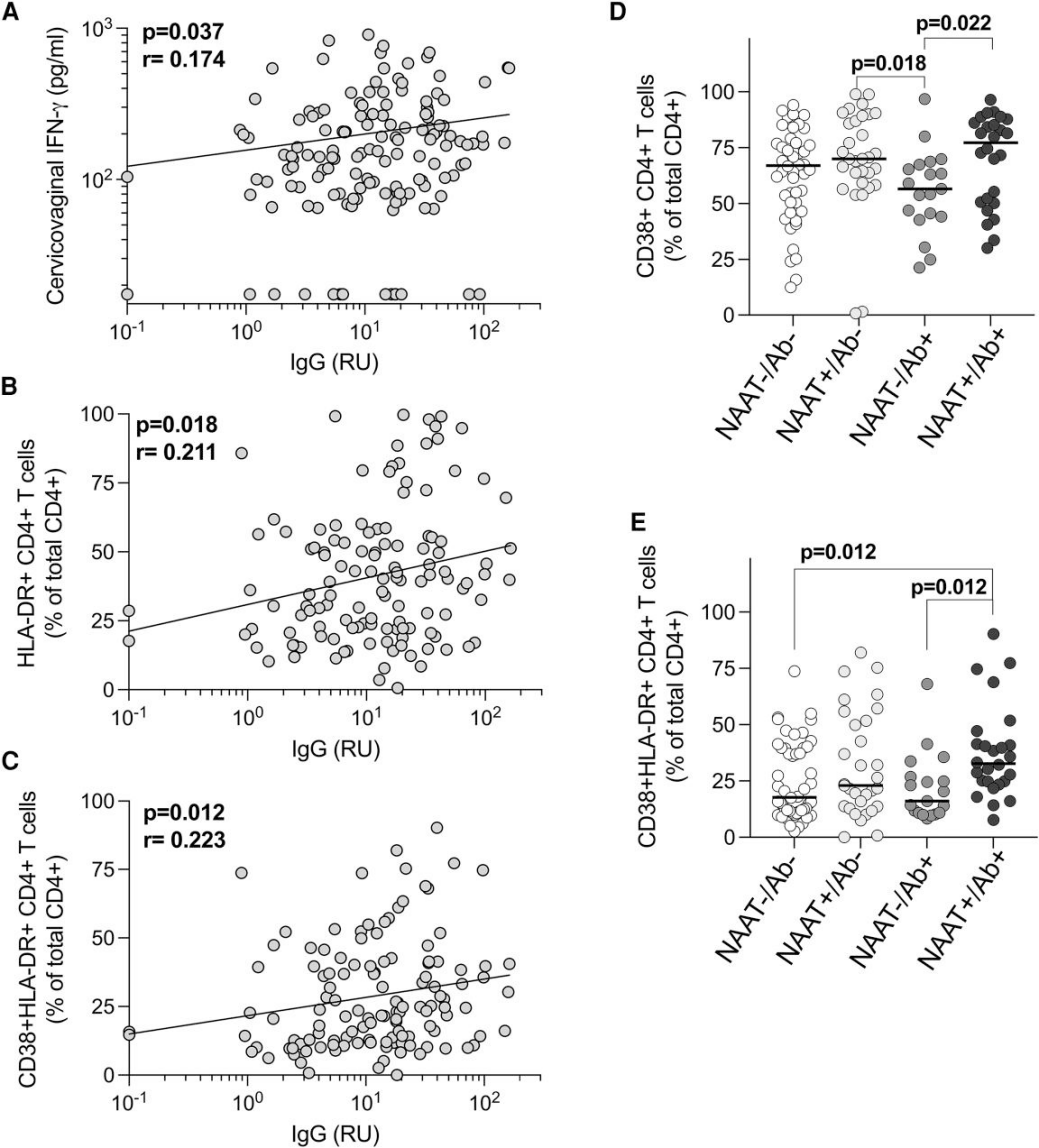
Exposure to *C. trachomatis* is associated with T cell activation in the female genital tract. Associations between IgG levels and (*A*) cervicovaginal IFN-γ concentrations (n = 144); and (*B*, *C*) frequencies of activated cervical T cells (n = 126). (*D*, *E*) Associations between relative exposure and the frequencies of T cells in the female genital tract (n = 126). Nonparametric Spearman correlations and Kruskal-Wallis statistical tests were performed. Statistical analyses were performed with false-discovery rate correction using the step-down procedure where appropriate. *P* < .05 was considered statistically significant. Ab = serostatus; NAAT = nucleic acid amplification test.

Previous work within this cohort showed that *C. trachomatis* infection was associated with elevated concentrations of genital tract inflammatory cytokines, including IL-6, TNF (also known as TNF-α), IL-1β, and IFN-γ [29]. Given that repeated or long-term exposure to *C. trachomatis* has been implicated in reproductive tract immunopathology [13], we examined relationships between exposure history, as measured by serology, and the immune environment in the genital tract. Antichlamydia IgG positively correlated with the concentration of cervicovaginal IFN-γ (*P* = .037, *r* = 0.174; Figure 2*A*), suggesting a significant but weak link between exposure and the mucosal IFN-γ response. No associations were observed between antibodies and other cytokines (IL-6, TNF, and IL-1β). Notably, antichlamydia IgG also positively correlated with the frequency of activated cervical CD4+ T cells expressing HLA-DR (*P* = .018, *r* = 0.211; Figure 2*B*) and those coexpressing CD38 and HLA-DR (*P* = .012, *r* = 0.223; Figure 2*C*). A similar association was seen with activated CD8 + HLA-DR + T cells (*P* = .019, *r* = 0.209; data not shown), though no correlations were found with CD4 + CD38+ T cells or CD8 + CD38+ T cell subsets alone.

We next compared cytokines and T cell activation across the relative exposure groups. While cytokine concentrations did not differ significantly between groups (data not shown), AGYW with evidence of prior, cleared *C. trachomatis* exposure (NAAT−/Ab+) had significantly lower frequencies of activated cervical CD4 + CD38+ T cells (median: 56.6%) compared to NAAT+/Ab− (*P* = .018; median: 70%) or NAAT+/Ab + (*P* = .022; median: 77.2%) participants (Figure 2*D*). Furthermore, highly exposed women (NAAT+/Ab+) had significantly greater frequencies of activated cervical CD4 + CD38 + HLA-DR + T cells compared to unexposed AGYW (medians: 32.7% and 17.75%, *P* = .018) or those with past infection (NAAT−/Ab+; medians: 32.7% and 16.1%, *P* = .022; Figure 2*E*), reflective of the active infection in the NAAT+/Ab + women. The associations between cumulative *C. trachomatis* exposure and elevated cervical T cell activation underscore the potential for immune-mediated pathology due to sustained antigenic persistence.

### Circulating *C. trachomatis*-Specific CD4+ T Cells Reflect Level of Exposure

**Figure 3.**
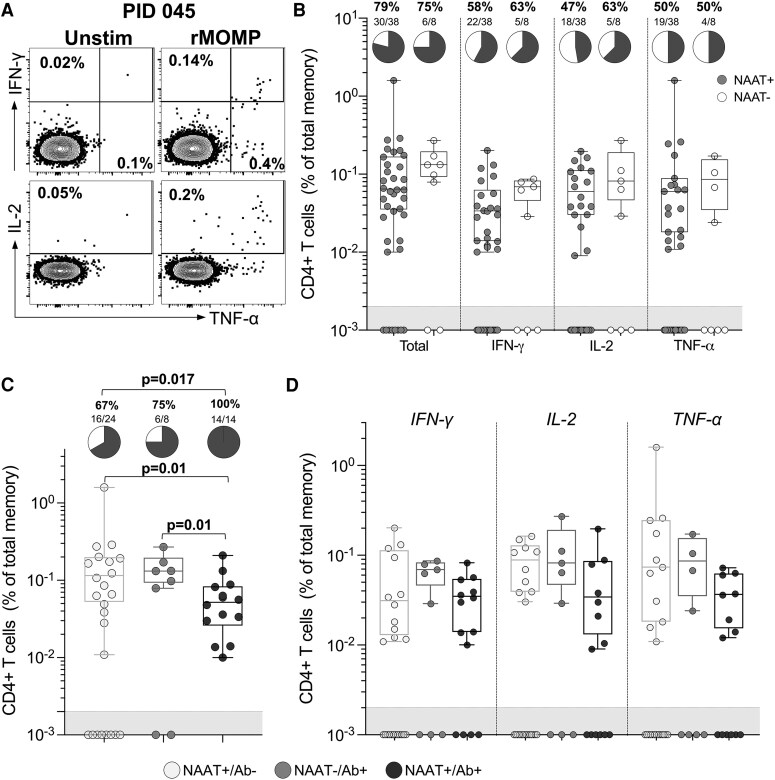
Peripheral CD4+ T cell responses to *C. trachomatis* are detectable in highly exposed AGYW. (*A*) Representative flow cytometry plots showing the IFN-γ, IL-2, and TNF response to rMOMP in one individual. (*B*) Frequency of CD4^+^ T cells responding to rMOMP in participants with a positive or negative NAAT result at baseline (n = 46). The number of participants with a positive cytokine response is represented by the pie charts. (*C*) Frequency of CD4^+^ T cells producing any cytokine in response to rMOMP across the 3 exposure groups (n = 46). Pie charts indicate the number of participants with a positive cytokine response, defined as twice the background value and a net response >0.005%. (*D*) *C. trachomatis-*specific CD4+ T cells producing IFN-γ, IL-2, or TNF. The box and whisker plots represent positive cytokine responses only. Each symbol represents an individual. Data were analyzed using a Kruskal-Wallis test. Fisher's exact test was used to determine differences between pie graphs. *P* < .05 was considered statistically significant. Ab = serostatus; NAAT = nucleic acid amplification test.

We measured *C. trachomatis*-specific CD4+ T cell cytokine responses in PBMCs) from AGYW who were either NAAT + or seropositive at baseline (n = 46; Figure 3*A*). Most women (n = 36/46) had detectable CD4+ T cell responses to the major outer membrane protein (rMOMP), regardless of NAAT status (Figure 3*B*). We also found no statistical differences in the proportion of NAAT− or NAAT + participants producing any cytokine (75% and 79%, respectively; *P* > .99), IFN-γ (63% and 58%, respectively; *P* > .99), IL-2 (63% and 47%, respectively *P* = .699) or TNF (50% for both; *P* > .99) in response to rMOMP (Figure 3*B*). Similarly, there were no statistical differences in the frequencies of *C. trachomatis*-specific CD4+ T cells producing any cytokine (median responses: 0.13% and 0.06%, respectively; *P* = .378), IFN-γ (median responses: 0.069% and 0.034%, respectively; *P* = .383), IL-2 (median responses: 0.082% and 0.06%, respectively; *P* = .321), or TNF (median of responses: 0.086% and 0.06%, respectively; *P* = .771; Figure 3*B*) between AAT + and NAAT− women. However, when participants were ranked by exposure, clear differences emerged. All the highly exposed AGYW (NAAT+/Ab+) had detectable CD4+ T cell responses (100%), compared to 67% of those with the lowest exposure (NAAT+/Ab-; *P* = .017). Furthermore, the magnitude of the T cell response was significantly lower in NAAT + Ab + women (median response: 0.05%) compared to NAAT+/Ab− (median response: 0.12%; *P* = .01) or NAAT−/Ab + women (median response: 0.13%; *P* = .01; Figure 3*C*), although this last group was very small (n = 8). There were no significant differences in cytokine-specific responses (*P* > .05; IFN-γ, IL-2, or TNF) across exposure groups (Figure 3*D*).

**Figure 4.**
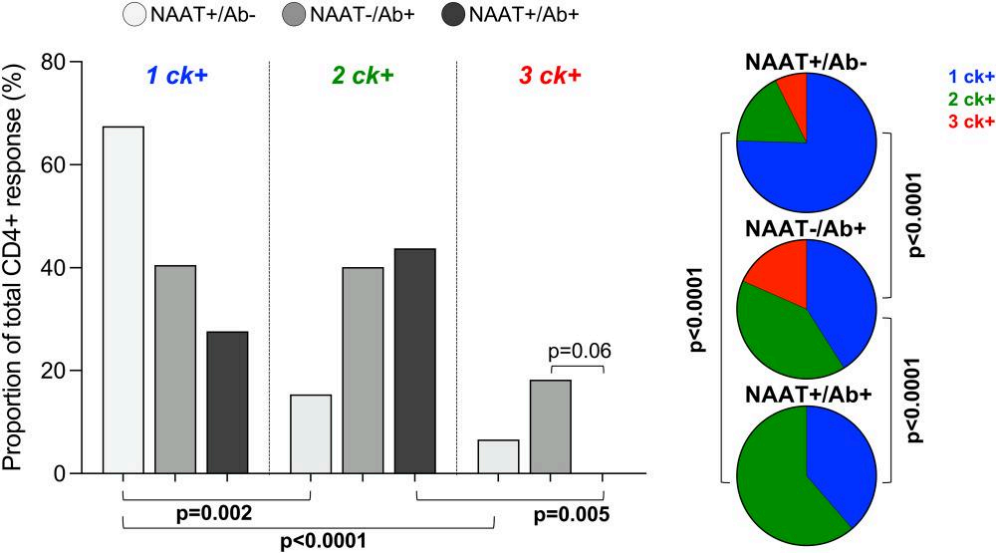
Exposure to *C. trachomatis* is reflected in CD4+ T cell function. The graph shows the proportion of *C. trachomatis-*specific CD4+ T cells producing different combinations of cytokines (n = 36). The pies show the polyfunctionality profiles of *C. trachomatis-*specific CD4+ T cells stratified by relative exposure (n = 36). Data shown are from participants with a positive total cytokine response. Each bar represents the median. Data were analyzed using a Kruskal-Wallis test. Fisher's exact test was used to determine differences between pie graphs. *P* < .05 was considered statistically significant. Ab = serostatus; NAAT = nucleic acid amplification test.

We next investigated the quality of the CD4+ T cell response to *C. trachomatis* by examining cytokine coexpression. Cytokine profiles were significantly different based on relative exposure (*P* < .0001; Figure 4). NAAT+/Ab− participants had predominantly monofunctional *C. trachomatis*-specific CD4+ T cells (median: 67.5%, IQR: 51–98.8%), and a low frequency of *C. trachomatis*-specific CD4+ T cells producing 3 cytokines (median: 6.62%, IQR: 0–19.9%). In contrast, NAAT−/Ab + AGYW had a more diverse response, with roughly equal proportions of cells producing 1 (medians and IQR: 40.5%; 15.9–86.3%) or 2 cytokines (40.2%; 9.1–45.4%), and a median of 18.2% (IQR: 4.6–35.4%) of cells producing all 3 measured cytokines. Interestingly, participants with the greatest exposure, NAAT+/Ab + lacked these highly functional T cells producing 3 cytokines (median: 0%, IQR: 0–5.9%) and showed a predominance of dual cytokine-producing cells (median: 43.8%, IQR: 21–100%). These findings suggest that while persistent or recurrent exposure to *C. trachomatis* increases the likelihood of detecting systemic T cell responses, it may also be associated with diminished functional quality, particularly a lack of cells coexpressing IFN-γ, IL-2, and TNF.

### *C. trachomatis*-specific T Cells are Associated With Reduced Genital Inflammation

**Figure 5.**
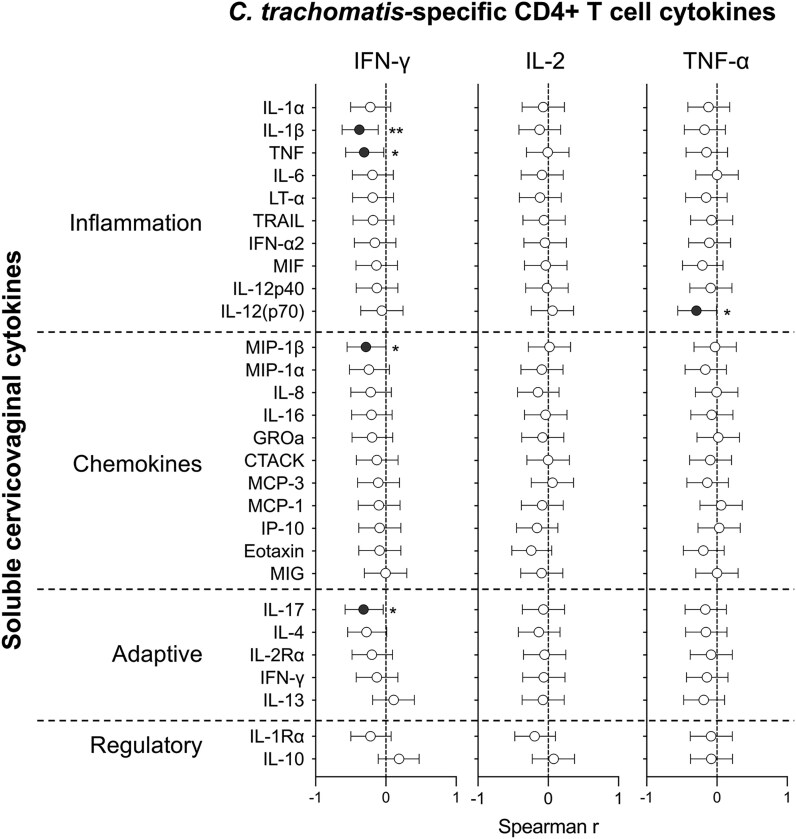
Associations between peripheral *C. trachomatis*-specific CD4+ T cell responses and the female genital tract immune milieu. Spearman correlation coefficient (*r*) and the 95% confidence interval are shown for associations between *C. trachomatis-*specific CD4+ T cells producing IFN-γ, IL-2, or TNF, and cervicovaginal cytokine concentrations (n = 22). Filled circles indicate statistically significant correlations. *P* < .05 was considered statistically significant. *P < .05; **P < .01; ***P < .001.

To understand how *C. trachomatis*-specific T cell immunity might influence pathogen-associated immunopathology in the genital tract, we examined relationships between peripheral CD4+ T cell responses and cervicovaginal cytokine concentrations in AGYW with matched data. Notably, the frequency of CD4+ T cells producing IFN-γ in response to *C. trachomatis* stimulation was inversely correlated with levels of several proinflammatory mediators in the genital tract, including IL-1β (*P* = .007, *r* = −0.397), TNF (*P* = .028, *r* = −0.328), MIP-1β (*P* = .045, *r*=−0.301), and IL-17A (*P* = .024, *r* = −0.337; Figure 5). Similarly, the frequency of *C. trachomatis*-specific CD4+ T cells producing TNF was significantly negatively associated with cervicovaginal IL-12p70 (*P* = .039, *r* = −0.308). Importantly, none of these mucosal cytokines were associated with exposure, suggesting that these correlations reflect antigen-specific immune regulation rather than cumulative exposure alone. There were no associations found with growth factors (data not shown).

## DISCUSSION

We examined circulating *C. trachomatis*-specific CD4+ T cells and their relationship with the genital tract immune milieu in women stratified into 3 groups based on increasing exposure: NAAT+/Ab−, NAAT−/Ab+, and NAAT+/Ab+, which we consider representative of primary infections, cleared/treated prior infections, and untreated/recurrent infections, respectively. Several important observations emerged from our study. The higher exposure to *C. trachomatis* in NAAT+/Ab + AGYW was associated with activated cervical T cells, consistent with previous studies describing an increased risk of inflammatory sequelae after recurrent *C. trachomatis* infection in response to persistent antigenic challenge [18, 38, 39]. In terms of *C. trachomatis*-specific T cell immunity, peripheral blood responses were detected in all NAAT+/Ab + women with untreated/recurrent infections, due to high antigen exposure. Notably, we also found evidence of defects in the CD4+ T cell response to *C. trachomatis* in this group. These highly exposed women had significantly lower frequencies of *C. trachomatis*-specific Th1 responses compared to women who were NAAT+/Ab− or NAAT−/Ab + . Furthermore, there was a near-total absence of highly functional *C. trachomatis*-specific CD4+ T cells simultaneously producing 3 cytokines in NAAT+/Ab + women. Meanwhile, NAAT−/Ab + AGYW had the highest proportion of these cells. These important and novel results emphasize that both the magnitude and quality of the CD4+ T cell response are vital for protective immunity to *C. trachomatis.*

Our findings may also reflect underlying T cell trafficking dynamics. In NAAT+/Ab + women, antigen-specific CD4+ T cells may have migrated from peripheral blood to the site of current infection, contributing to local immunity. Supporting this, murine studies have shown that *C. trachomatis*-specific T cells producing IFN-γ circulate between the genital tract and peripheral blood [40]. Clinical studies have similarly reported elevated IFN-γ concentrations in the female genital tract during recurrent *C. trachomatis* infections [29, 41]. Here, cervicovaginal IFN-γ concentrations correlated with antichlamydia IgG titers, supporting its use as a marker of cumulative exposure. Given the well-established role of IFN-γ in pathogen clearance, we hypothesize that cells producing IFN-γ, including *C. trachomatis*-specific Th1 cells, contribute to protective immunity at mucosal surfaces. Consistently, we found significant negative associations between the frequency of peripheral *C. trachomatis*-specific CD4+ T cells and concentrations of cervicovaginal inflammatory mediators like IL-1β and TNF, suggesting a possible reduction in potential immunopathology when higher circulating antigen-specific T cells are detected. These findings suggest that systemic *C. trachomatis*-specific Th1 responses may not only reflect effective immune control but could also contribute to the regulation of immunopathology. Although technically challenging, *C. trachomatis*-specific T cells should be studied at the site of infection to fully disentangle the development of protective immunity and immunopathology.

Earlier studies sought to identify correlates of protection from subsequent reinfection following treatment. Bakshi et al. [42] demonstrated that *C. trachomatis*-specific CD4+ IFN-γ T cell responses were largely absent in women who were reinfected, compared to those who were not. Although the differences in our studies preclude direct comparison, we found that women with untreated/recurrent infections (NAAT+/Ab+) had fewer highly functional CD4+ T cells, despite a high prevalence of detectable responses. This lack of functional diversity may reflect T cell exhaustion or impaired differentiation, processes commonly associated with chronic antigenic stimulation in other infectious settings [43].

We classified participants into exposure groups using baseline NAAT and serology to gain a more nuanced understanding of *C. trachomatis* infection history. There were some caveats to this approach. Since the treatment data was incomplete, we cannot infer the duration of current or past infections. The short time since sexual debut and the documented persistence of antichlamydia antibodies led us to classify NAAT+/Ab− as primary infections prior to the development of antibodies [35]. However, we cannot entirely rule out failure to seroconvert, rather than early infection. Thus, future studies should also measure IgM, since IgM seroconversion remains poorly characterized [44]. Nevertheless, these groupings still reflect the relative level of *C. trachomatis* exposure and provide valuable insights into chronic antigen exposure and immunity. A limitation of this study was that NAAT was only performed on vulvovaginal swabs, potentially missing anorectal infections [45], which would affect the classification of NAAT− participants.

We assessed T cell responses using a highly immunogenic recombinant protein antigen, the major outer membrane protein [46]. Although recombinant proteins have the potential to activate nonantigen-specific T cells, a short-term assay was used specifically to minimize this effect. While we cannot completely exclude the possibility of nonspecific stimulation, the immune response to rMOMP, including some degree of nonspecific activation, remains biologically relevant, as in vivo immune responses would similarly involve both antigen-specific and nonspecific T cell activation.

*C. trachomatis-specific* Th1 responses in blood occur at low frequencies, making them difficult to consistently detect in humans, depending greatly on assay conditions [25, 45]. Our findings need to be validated in larger cohort studies, with additional antigens, eg, heat-shock protein 60, or chlamydial protease-like activity factor (CPAF) to capture a greater breadth of the response [47]. Mucosal-acting cytokines like IL-17A or immunoregulatory cytokines such as IL-10 may also be important in the response to *C. trachomatis.* Indeed, previous work reported that women who remained uninfected had higher frequencies of bulk Th17 cells, suggesting a role in protection [48]. The ability to identify robustly detectable responses, ideally in blood, will be crucial for future evaluation of vaccine-induced immunity and potential correlates of protection.

In conclusion, we showed that women with untreated or recurrent infections had lower frequencies of *C. trachomatis*-specific CD4+ T cells, and a smaller proportion of these cells were highly functional. Furthermore, peripheral T cell responses negatively correlated with inflammation in the genital tract. Our data clearly indicate that both the magnitude and function of the CD4+ T cell immune response to *C. trachomatis* are important components of protective immunity. These findings underscore the importance of including mucosal immune markers and functional systemic responses in the evaluation of candidate chlamydia vaccines, especially in high-risk populations such as AGYW.

## Supplementary Material

jiaf595_Supplementary_Data

## Supplementary Data

Supplementary materials are available at *The Journal of Infectious Diseases* online (http://jid.oxfordjournals.org/). Supplementary materials consist of data provided by the author that are published to benefit the reader. The posted materials are not copyedited. The contents of all supplementary data are the sole responsibility of the authors. Questions or messages regarding errors should be addressed to the author.
