# Supplementary material for: Chlamydia trachomatis-specific T Cell Immunity Reflects Widespread Exposure in South African Adolescents and Young Women
Source: J Infect Dis. 2025 Dec 3;233(2):e290–300. doi: 10.1093/infdis/jiaf595 (PMC13017485; doi:10.1093/infdis/jiaf595)
Supplement: jiaf595_Supplementary_Data [file jiaf595_supplementary_data.pdf]

Table S1: *C. trachomatis* serostatus and exposure classification

Characteristic [n (%)]	N=145
Serostatus	
Seropositive	*44 (30%)*
Seronegative	*81 (56%)*
Borderline	*20 (14%)*
Exposure	
Uninfected [NAAT-; seronegative]	*60 (41%)*
Primary infection [NAAT+; seronegative/borderline]	*33 (23%)*
Cleared infection [NAAT-; seropositive/borderline]	*23 (16%)*
Untreated/recurrent infection [NAAT+; seropositive]	*29 (20%)*

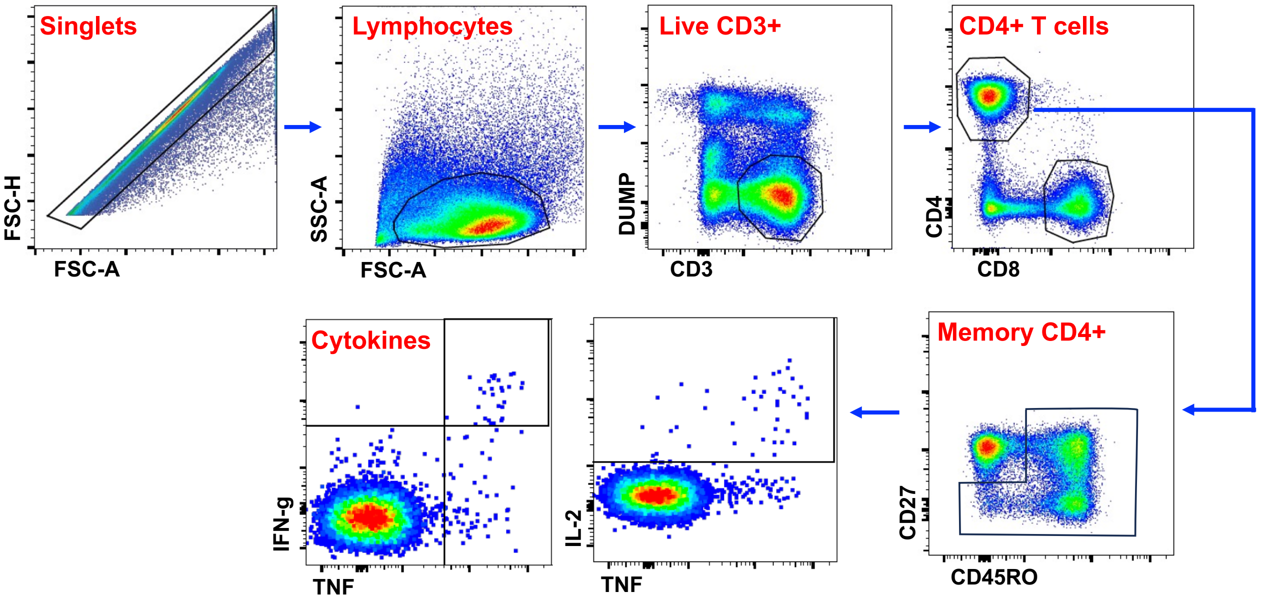

Figure S1: Gating strategy for identifying antigen-specific cells. Time gates were drawn for one channel on each laser, followed by singlets, lymphocytes, live CD3+, CD4+CD8- cells. After positively identifying all memory CD4+ T cells, cytokine gates were drawn and set consistently between stimulated and unstimulated samples.
